# Is Extracorporeal Membrane Oxygenation a Panacea?

**DOI:** 10.5152/eurasianjmed.2023.23269

**Published:** 2023-12-01

**Authors:** Selen Karaoğlanoğlu, Metin Akgün

**Affiliations:** 1 Department of Pulmonology, Ordu University Faculty of Medicine, Ordu, Turkey; 2 Department of Pulmonology, Ağrı İbrahim Çeçen University Faculty of Medicine, Ağrı, Turkey

**Keywords:** ARDS, COVID-19, ECPR, extracorporeal life support

## Abstract

Extracorporeal membrane oxygenation (ECMO) has emerged as a vital life-support technique in critical care medicine, providing temporary circulatory and/or respiratory support for patients with severe cardiac or respiratory failure unresponsive to conventional therapies. This review aims to outline the importance of ECMO and provide a comprehensive overview of its main applications. Two primary types of ECMO, veno-arterial extracorporeal membrane oxygenation and veno-venous extracorporeal membrane oxygenation, serve distinct functions in supporting patients with cardiac or pulmonary dysfunction, respectively. While ECMO offers life-saving potential, its utilization requires careful consideration due to its cost and resource-intensiveness. Thus, a comprehensive evaluation of an individual patient’s clinical condition, prognosis, and potential for recovery is crucial. Ongoing research and technological advancements continually refine ECMO techniques, enhance patient selection criteria, and improve long-term outcomes. Within this narrative review, we present an updated approach to patient selection and ECMO utilization, supported by a detailed literature review. By consolidating the current evidence, we aim to provide healthcare professionals with valuable insights into the ECMO’s post-pandemic role.

**Main Points**

- We wrote this review to highlight the following main headings:

- Extracorporeal membrane oxygenation (ECMO) is a life-saving medical intervention used in critical care, but it is not a universal cure-all solution.
- Extracorporeal membrane oxygenation provides mechanical support to patients with severe respiratory or circulatory failure when conventional treatments fail.
- While ECMO has shown remarkable success in certain cases, its efficacy varies depending on patient selection, timing, and underlying conditions.
- The decision to initiate ECMO should be carefully considered, and its benefits should be weighed against potential complications, including bleeding and infection.
- Extracorporeal membrane oxygenation’s role in critical care highlights the need for a multidisciplinary approach, ongoing research, and strict protocols to optimize its use and improve patient outcomes.

## Introduction

Mechanical cardiopulmonary support is commonly employed during both intraoperative and intensive care settings, particularly in cardiac surgery. However, sometimes cardiopulmonary support may be required in the intensive care unit. Extracorporeal membrane oxygenation (ECMO) or extracorporeal lung support (ECLS) serves as a life support system for providing prolonged cardiopulmonary assistance. Veno-arterial extracorporeal membrane oxygenation (VA-ECMO) and veno-venous extracorporeal membrane oxygenation (VV-ECMO) are 2 primary types of ECMO, offering respiratory support, with VA-ECMO additionally providing hemodynamic support. Since its inception in 1970, the ECMO experience has progressively expanded, gaining significant recognition.^1,2^ In this review, we comprehensively discuss the clinical advantages of ECMO, patient selection criteria, indications, contraindications, and its specific role in managing respiratory failure associated with viral pneumonia, which has become increasingly prevalent during the coronavirus disease 2019 (COVID-19) pandemic.^3^ Key studies are summarized in Table 1, and by incorporating an extensive review of available literature, our aim is to provide valuable insights and guidance for clinical practice in this evolving field.

Notably, neonates and children generally exhibit high survival rates following ECMO support for respiratory failure.^4^ Nevertheless, accurately estimating patient survival poses a challenge, as the mortality risk associated with this procedure is estimated to be approximately 50%. Typically, ECLS is considered when the risk of mortality reaches around 80%. To ensure a precise evaluation, the severity of the disease, likelihood of death, and organ failure are meticulously assessed, considering patient age and other factors.^5^ Complications may arise, some of which may result in significant morbidity. Extracorporeal membrane oxygenation-associated complications can be categorized as either device related, encompassing issues such as oxygenator malfunction, pump failure, circulatory blockages, and cannulation problems, or physiological, including bleeding, hemolysis, and infection.^6-9^ Initiation of ECMO triggers an inflammatory response akin to systemic inflammatory response syndrome.^10^ The interaction between patient blood and the extracorporeal space initiates coagulation and inflammatory cascades, rapidly elevating proinflammatory cytokine levels and activating leukocytes.^11-14^ This innate immune response ultimately leads to endothelial damage, impaired microcirculation, and subsequent end-organ dysfunction.^10-15^ Despite the escalating use of ECMO, our current understanding of the elicited inflammatory response remains limited. Patients supported with ECMO frequently exhibit an inflammatory response; however, a comprehensive understanding of the severe patient reactions to inflammation and their clinical trajectory is currently lacking. Further insight into this complex phenomenon is necessary to explore potential treatments and novel therapeutic strategies.

## Techniques of Extracorporeal Membrane Oxygenation

**Figure 1.**
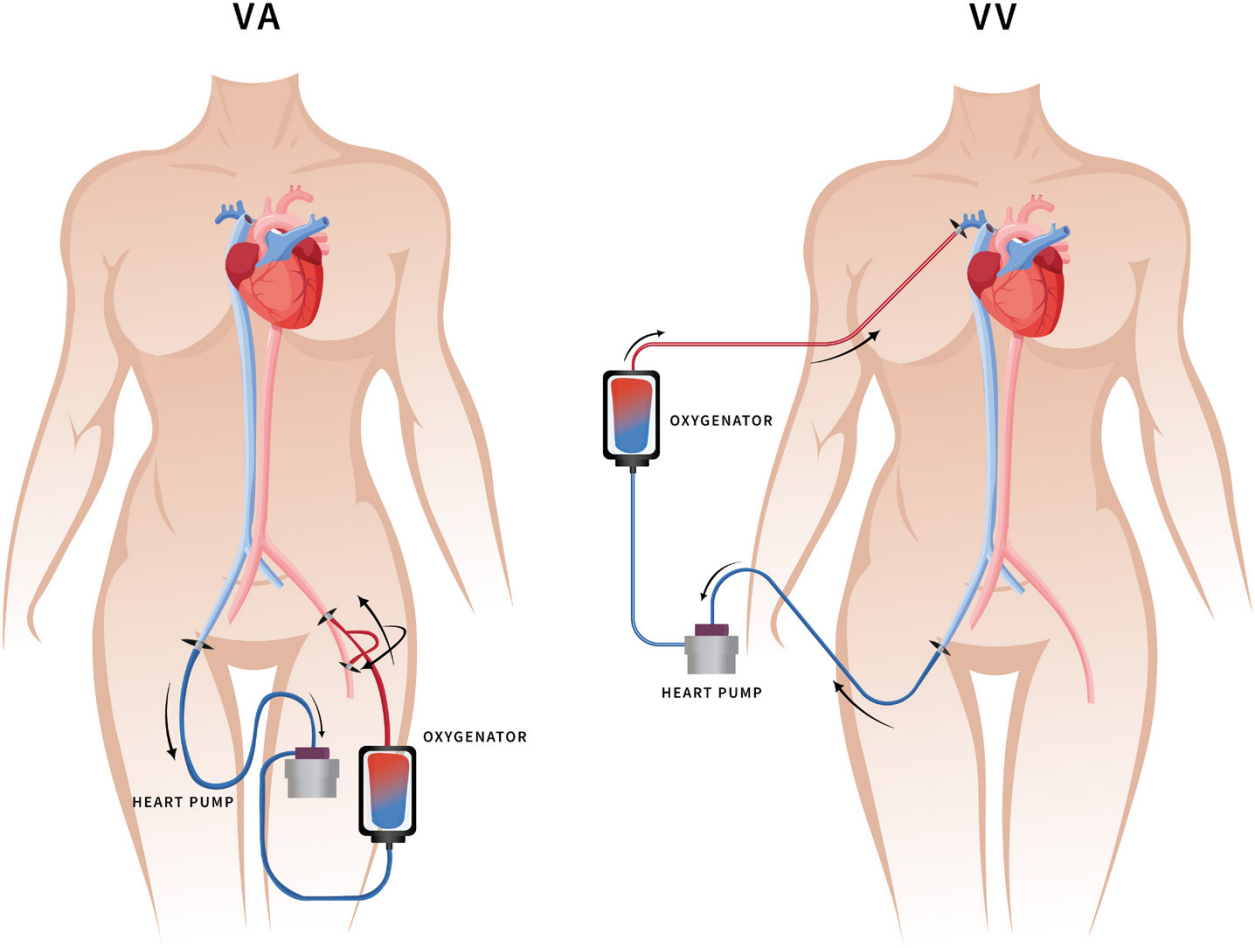
The figure shows the 2different types of extracorporeal membrane oxygenation, the veno-atrial (VA) and the veno-venous (VV) systems.

Two ECMO techniques are commonly used (Figure 1):

1. Veno-venous ECMO: It provides respiratory support by removing deoxygenated blood from a vein, oxygenating it, and returning it to a vein. This configuration bypasses the heart and supports the lungs.^5^
  - Cannulation: In VV-ECMO, 2 cannulas are typically inserted into a large central vein, such as the femoral vein or internal jugular vein. The venous cannula drains deoxygenated blood from the patient, which is then advanced into the right atrium or superior vena cava. Oxygenated blood is returned to the patient via a second cannula, usually placed in a central vein, such as the internal jugular vein or subclavian vein.
  - Circuit: The cannulas are connected to the ECMO circuit, which includes a pump, a membrane oxygenator, and tubing. The pump propels blood through the circuit, while the membrane oxygenator removes carbon dioxide and adds oxygen. Prior to returning it to the patient, the oxygenated blood is warmed to body temperature.
2. Veno-arterial ECMO: It provides both respiratory and cardiac support by bypassing both the heart and lungs.
3. Cannulation: Similar to VV-ECMO, 2 cannulas are used in VA-ECMO. The arterial cannula is inserted into a large artery, such as the femoral artery, to withdraw deoxygenated blood from the patient. The cannula is advanced to a site distal to the heart. Oxygenated blood is then returned to the patient via a venous cannula, as in VV-ECMO.
4. Circuit: The ECMO circuit for VA-ECMO is similar to VV-ECMO, consisting of a pump, a membrane oxygenator, and tubing. The pump propels blood through the circuit, providing oxygenation and mechanical support to both the heart and lungs. The oxygenated blood is warmed to body temperature and returned to the patient via the venous cannula.^16,17^

## Hemostasis of Extracorporeal Membrane Oxygenation

Effective hemostasis management is a crucial aspect of ECMO therapy, as the use of anticoagulation to prevent clot formation within the circuit presents challenges in maintaining hemostasis. Achieving the delicate balance between preventing bleeding and minimizing thrombotic complications remains a constant concern. Close monitoring of coagulation parameters, such as activated clotting time or activated partial thromboplastin time, is essential for guiding anticoagulation therapy and detecting early signs of bleeding or clotting. Collaborative efforts among the ECMO team, hematologists, and other specialists are vital to develop personalized hemostasis management strategies. These strategies may involve adjusting anticoagulation levels, employing antithrombotic agents, and implementing techniques to minimize circuit-related factors that contribute to coagulation activation. The goal is to maintain optimal hemostasis, prevent both bleeding and thrombotic complications, and ultimately improve patient outcomes during ECMO therapy.^18^

## Selecting the Perfect Candidate for Extracorporeal Membrane Oxygenation

Extracorporeal membrane oxygenation, a transformative technology with immense potential to save lives, necessitates cautious implementation to prevent additional harm to patients. Integration of ECMO within comprehensive clinical strategies, such as long-term platforms like ventricular assist devices and transplantation, becomes imperative, as it is not a viable standalone solution.^19^ The survival rates for patients who suffer out-of-hospital cardiac arrest (OHCA) remain alarmingly low. However, the use of ECMO in cases of cardiac arrest, known as extracorporeal cardiopulmonary resuscitation (ECPR), has demonstrated promising results in enhancing patient survival while preserving favorable neurological outcomes.^20^ A Danish study revealed a substantial failure rate, with only a minority of patients undergoing ECPR treatment achieving successful outcomes. Factors contributing to the avoidance of ECPR encompass prolonged prehospital low-flow duration, metabolic abnormalities, and diminished end-tidal carbon dioxide (ETCO_2_) levels.^21^

## Indications

Patient selection and timing are crucial considerations in ECMO, with mortality rates rising with age and concomitant diseases.^22,23^ The use of scoring systems such as Respiratory ECMO Survival Prediction (RESP) and Murray scores can assist in the assessment. The RESP score predicts survival for ECMO patients, while the Murray score predicts mortality rates in the absence of ECMO. If ECMO is deemed necessary, prompt transfer to a specialized medical facility should be arranged.^24^ Clinical guidelines for Acute Respiratory Distress Syndrome (ARDS) recommend considering ECMO in adult patients with severe ARDS, supported by a moderate level of evidence (GRADE rating 2B). There are many experimental studies in the treatment of ARDS.^25,26^ Patients with severe hypoxemia or hypercapnia who do not respond to conventional lung-protective ventilation or adjunctive therapies should be referred to experts for guidance on ECMO indications and patient transport.^27^ Survival rates range from 50% to 71%.^28-33^ The CESAR study compared ECMO with standard ventilatory support in severe acute respiratory failure, revealing significantly higher 6-month survival without sequelae (63% vs. 47%) with ECMO, despite certain methodological limitations, including the study being conducted at a single center with specialized expertise in ECMO use.^34^ In the EOLIA study, early VV-ECMO versus delayed intervention in ARDS showed no statistically significant difference in 60-day mortality rates.^35^ While the appropriateness of ECMO in ARDS remains debated, studies suggest potential benefits despite challenges in conducting controlled studies in critically ill patients.^34-36^ Veno-venous ECMO is recommended for adult patients with severe ARDS due to sepsis and failed mechanical ventilation, although quality of the evidence is low. Studies in England^37^ and France^38^ reported varied mortality rates for severe acute respiratory failure associated with influenza H1N1 patients transferred to ECMO centers. Extracorporeal membrane oxygenation may serve as a bridge to transplantation, benefiting some patients with chronic respiratory failure from interstitial lung disease.^39^ Evidence supporting VA-ECMO use for circulatory assistance in cardiogenic shock remains inadequate, as the ECMO-CS trial found no significant differences in clinical outcomes between immediate initiation and a conservative approach with delayed use.^40^

Recent randomized trials suggest potential benefits of ECPR, yet evidence certainty is modest, and patients’ selection criteria remain undetermined.^41^ Extracorporeal cardiopulmonary resuscitation in intra-hospital cardiac arrest (IHCA) shows low survival rates but favorable neurological outcomes at 1 year.^42^ Variables such as age, time of day, initial rhythm, medical history of renal failure, patient type (cardiac versus noncardiac and medical versus surgical), and duration of cardiac arrest influence IHCA causes and survival rates.^43^ Extracorporeal cardiopulmonary resuscitation is more effective in OHCA,^44^ with early chest compression initiation improving pre-discharge rates.^45^ Implementing transportable VA-ECMO programs in hospitals allows treatment of critically ill patients with comparable survival rates.^46^ Predictors of survival in OHCA patients include initial cardiac rhythm, short symptom onset-to-arrival duration, and age below 75 years.^47-49^ Out-of-hospital ECPR has been shown to be an effective^50^ and economically acceptable pacing strategy.^51^ Accidental cardiac arrest (AHCA) is another indication for ECLS.^52-56^ Extracorporeal membrane oxygenation improves outcomes in AHCA cases, especially in acute hypothermia-induced cardiac arrest.^57^ Veno-arterial ECMO and endovascular therapy are rescue strategies for massive pulmonary embolism (PE) when thrombolysis is contraindicated, potentially aiding resuscitation in high-risk PE-related cardiac arrest.^58,59^ Immediate initiation of ECMO can potentially aid in resuscitation of patients with cardiac arrest due to high-risk PE.^60,61^ Veno-arterial ECMO and endovascular therapy are rescue strategies for massive PE when thrombolysis is contraindicated, potentially aiding resuscitation in high-risk PE-related cardiac arrest (57, 58). Successful VA-ECMO use has been reported in amniotic fluid embolism cases.^62,63^ Extracorporeal cardiopulmonary resuscitation indications in cancer patients are unclear, but understanding IHCA outcomes in this population is essential due to the incidence of cancer and improved survival rates.^64^ Veno-arterial ECMO is a suitable treatment option for poisoning cases complicated by refractory cardiogenic shock or cardiac arrest, offering high survival rates with low complications.^65^ Extracorporeal membrane oxygenation may be indicated in severe status asthmaticus,^66^ pheochromocytoma-induced cardiomyopathy,^67,68^ fulminant myocarditis,^69^ ANCA-associated vasculitis,^70^ and refractory thyroid storm.^71^

## Contraindications

Relative contraindications to ECMO include age older than 65 years, body mass index greater than 40, suppressed immunity, lack of a relative who can make medical decisions, and severe chronic systolic heart failure. There are certain contraindications to ECMO that should be considered, such as disseminated malignancy, severe deconditioning, uncontrolled bleeding, inability to receive blood products, ongoing CPR, third-stage chronic renal disease, severe peripheral vascular disease, uncontrolled diabetes with chronic end-organ dysfunction, cirrhosis, advanced lung disease, dementia, other preexisting life-limiting conditions, a clinical frailty scale of category 3 or higher, underlying neurologic disease affecting rehabilitation potential, severe multiorgan failure, and advanced age.^5,72,73^

## Complications of Extracorporeal Membrane Oxygenation

Complications of ECMO are significant, as observed in an analysis of the Extracorporeal Life Support Organization (ELSO) involving 7579 VV-ECMO patients. Among them, 40.2% experienced bleeding and thrombotic events, with thrombosis in the ECMO circuit being predominant. Hospitalized patients had a reported mortality rate of 34.9%. Thrombotic events (adjusted OR 1.23; 95% CI, 1.08-1.41; *P* < .01) and bleeding events (adjusted OR 1.69, 95% CI, 1.49-1.93; *P* < .01) were differentially associated with in-hospital mortality. Ischemic stroke, intracranial hemorrhage, pulmonary hemorrhage, and gastrointestinal hemorrhage were strongly linked to mortality (adjusted ORs ranging from 2.02 to 5.71, all *P* < .01). Bleeding and thrombosis were associated with prolonged ECMO duration, younger age, elevated pH levels, and earlier years of support.^74^ Disseminated intravascular coagulation has been reported in adult ECMO patients, often related to preexisting conditions such as liver failure, poor anticoagulation mechanisms, and increased fibrinolysis.^75^

## Extracorporeal Membrane Oxygenation and Coronavirus Disease 2019

In March 2020, the World Health Organization (WHO) declared the COVID-19 pandemic, which has had a widespread global impact, affecting over 180 million people and resulting in approximately 6 million deaths.^76-79^ During the pandemic, many studies were conducted to early determine the prognosis and intensive care needs of patients.^80-89^ In response to the escalating pandemic, ECMO use has substantially increased. As of the time of writing this review, approximately 6390 COVID-19 patients were reported to have received ECMO assistance.^90^ Guidelines from the Chinese National Health Commission recommended the use of ECMO in the prone position when necessary and respiratory support for the treatment of severe and critically ill patients with lung healing status.^91^ Transitional guidelines by ELSO for COVID-19 state that as the burden of disease increases, ECMO capacity will expand to benefit those who can regain an acceptable quality of life. In regions with crowded hospitals, early transfer of appropriate ECMO candidates, particularly young individuals with single organ failure and no preexisting health conditions, to ECMO centers is prudent. Specific criteria, such as the PaO_2_/FiO_2_ ratio, pH level, and PaCO_2_ values, help determine ECMO indications.^92^ Extracorporeal membrane oxygenation may also be indicated for COVID-19-related cardiogenic shock and refractory cardiac arrest due to myocarditis.^93^ In cases of refractory cardiogenic shock characterized by persistent tissue hypoperfusion and inadequate cardiac function, the timely initiation of VA-ECMO before multiorgan failure occurs is recommended. The need for VA-ECMO is generally uncommon but may be considered as an adjunct treatment for failed cardiogenic/obstructive shock in patients with ARDS, acute stress/septic cardiomyopathy, or in cases of massive PE.^92^ A systematic review examining the use of mechanical circulatory support (MCS) in COVID-19 patients revealed that out of 4218 individuals, 92.7% received VV-ECLS, 4.7% received VA-ECLS and/or Impella, and 2.6% received other forms of ECLS. Conversion from VV-ECLS to MCS was necessary for 3.1% of patients due to heart failure, myocarditis, or myocardial infarction. Survival rates for VV-ECLS and MCS were reported as 54.6% and 28.1%, respectively.^94^ Another study evaluated the effectiveness of the RESP score (Table 2) in predicting in-hospital survival in COVID-19 patients receiving VV-ECMO. However, the exclusive use of the RESP score was deemed insufficient for accurately predicting survival in COVID-19 patients requiring VA-ECMO treatment. Further investigation is needed to determine the most appropriate timing and indication for MCS in COVID-19 patients based on survival reports.^95^ Considering the potential for neurological injury, additional research is necessary to explore neuromonitoring protocols to enhance personalized anticoagulation management and improve survival rates in COVID-19 patients on ECMO.^96^ Given resource limitations, ECMO should be reserved for extremely severe cases of COVID-19 during a global pandemic.^97^ The utility of ECMO should be evaluated based on current circumstances, considering the need for specialized patient care and the capacity to manage a high volume of patients through centralization.^98^

## Conclusion

The utilization of VV-ECMO in the adult population has witnessed a significant global increase. Careful patient selection is paramount, ensuring that the etiology of respiratory failure in ARDS is reversible and unresponsive to conventional treatments while also considering formal contraindications to ECMO initiation. Furthermore, patients with irreversible diseases, such as end-stage lung disease, may be considered viable candidates for ECMO therapy, particularly when bridging toward lung transplantation. The employment of ECMO in the management of ARDS patients has demonstrated superior survival rates when compared to patients of similar age and disease severity who did not receive this advanced intervention. Nevertheless, it is noteworthy that the current body of literature addressing the use of VA-ECMO to support COVID-19 patients is sparse, necessitating further investigation to optimize its application and elucidate its potential benefits in this specific context.

## Figures and Tables

**Table 1.** Respiratory Extracorporeal Membrane Oxygenation Survival Prediction Score Values for Calculation

Characteristics	Score
Age (years)	
18-49	0
50-59	−2
≥60	−3
Mechanical ventilation (hours)	
0-47	3
48-167	1
≥168	0
Cardiac arrest before ECMO	−2
PaCO_2_, mmHg	
<75	0
≥75	−1
Peak inspiratory pressure, cmH_2_O	
<42	0
≥42	−1
Immunocompromised	−2
CNS* dysfunction	−7
Neuromuscular blocker before ECMO	1
Nitric oxide before ECMO	−1
Bicarbonate before ECMO	−2
Viral pneumonia (COVID-19 positive)	3
Total RESP score	**Risk Class**
≥6	I
3-5	II
From −1 to 2	III
From −5 to −2	IV
≤ −6	V

ECMO, extracorporeal membrane oxygenation; *CNS, Central Nervous System; RESP, respiratory ECMO survival prediction.

**Table 2.** Literature Overview: Extracorporeal Life Support (Extracorporeal Membrane Oxygenation) Techniques, Indications, Contraindications, Complications, and Coronavirus Disease 2019 Considerations

Author(s)	Year	Title	Main Points
**ECMO Techniques**			
Bartlett et al^16^	2010	Current Status of Extracorporeal Life Support (ECMO) for Cardiopulmonary Failure	ECMO provides temporary life support for patients with cardiopulmonary failure, allowing time for diagnosis, treatment, and recovery.
Gabelloni et al^17^	2022	ECMO pocket guide for radiologists	Correct placement of ECMO cannulae and imaging features of potential complications and disease evolution in COVID-19 patients treated with ECMO.
**Hemostasis of ECMO**			
McMichael et al^18^	2022	ELSO Adult and Pediatric Anticoagulation Guidelines	These guidelines provide educational information for healthcare professionals regarding anticoagulation during ECMO, emphasizing the importance of individual judgment and patient consultation.
**Selecting the Perfect Candidate for ECMO**			
Tonna et al^19^	2021	Management of Adult Patients Supported with Veno-venous Extracorporeal Membrane Oxygenation (VV-ECMO): Guideline from the Extracorporeal Life Support Organization (ELSO)	The global utilization of VV-ECMO in adults has experienced a swift proliferation. Hence, this ELSO guideline aims to serve as a pragmatic manual encompassing patient selection, initiation, cannulation, management, and weaning strategies for VV-ECMO in cases of adult respiratory failure.
Linde et al^21^	2023	Selection of patients for mechanical circulatory support for refractory out-of-hospital cardiac arrest	This study aims to describe patient characteristics in refractory cardiac arrest cases and explore reasons for refraining from ECPR treatment, providing insights into patient selection. Factors such as prolonged prehospital low-flow time, metabolic derangement, and low ETCO_2_ were common reasons for abstaining from ECPR.
**Indications of ECMO**			
Banavasi et al^34^	2021	Management of ARDS - What Works and What Does Not	ECMO should be considered for selected severe ARDS patients undergoing lung-protective ventilation, based on criteria such as a Murray Score >3 or pH <7.2 due to uncorrected hypercapnia. Additionally, factors like age, medical conditions, underlying ARDS causes, and ECMO availability should also be taken into account.
Huang et al^23^	2020	Clinical features of patients infected with 2019 novel coronavirus in Wuhan, China	This study reports the epidemiological, clinical, laboratory, and radiological characteristics and treatments and clinical outcomes of a cluster of pneumonia cases in Wuhan, China.
Peek et al^32^	2009	Efficacy and economic assessment of conventional ventilatory support versus extracorporeal membrane oxygenation for severe adult respiratory failure (CESAR): a multicentre randomized controlled trial	Adult patients with potentially reversible severe respiratory failure should be transferred to a specialized center with an ECMO-based management protocol. This recommendation applies to patients with a Murray score > 3.0 or pH level < 7.2 despite optimal conventional treatment. The goal is to improve survival rates and reduce the risk of severe disability.
Combes et al^33^	2018	Extracorporeal Membrane Oxygenation for Severe Acute Respiratory Distress Syndrome	Among patients with very severe ARDS, 60-day mortality was not significantly lower with ECMO than with a strategy of conventional mechanical ventilation that included ECMO as rescue therapy.
**Contraindications of ECMO**			
Extracorporeal Life Support Organization (ELSO)^5^	2017	Guidelines for Cardiopulmonary Extracorporeal Life Support Organization	This is a general guideline that consists of all indications and contraindications
Schmidt et al^71^	2015	Predicting survival after ECMO for refractory cardiogenic shock: the survival after veno-arterial-ECMO (SAVE)-score	The study suggests that chronic renal failure, longer duration of ventilation prior to ECMO initiation, pre-ECMO organ failures, pre-ECMO cardiac arrest, congenital heart disease, lower pulse pressure, and lower serum bicarbonate (HCO_3_) were risk factors associated with mortality.
Schmidt et al^72^	2013	The PRESERVE mortality risk score and analysis of long-term outcomes after extracorporeal membrane oxygenation for severe acute respiratory distress syndrome	The PRESERVE score might help ICU physicians select appropriate candidates for ECMO among severe ARDS patients.
**Complications of ECMO**			
Nunez et al^73^	2022	Bleeding and thrombotic events in adults supported with veno-venous extracorporeal membrane oxygenation: an ELSO registry analysis	Bleeding risk factors encompass acute kidney injury and the administration of vasopressors prior to ECMO. Conversely, thrombosis risk factors include increased weight, multiple cannulation sites, previous arrest before ECMO, and elevated PaCO_2_ levels at the start of ECMO. Prolonged duration on ECMO, younger age, higher pH levels, and earlier years of support are associated with both bleeding and thrombosis incidents.
**ECMO and COVID-19**			
Shekar et al^78^	2020	Extracorporeal Life Support Organization Coronavirus Disease 2019 Interim Guidelines: A Consensus Document from an International Group of Interdisciplinary Extracorporeal Membrane Oxygenation Providers	Support the current ECMO facilities in their efforts to get ready and strategize for the provision of ECMO treatment amid the ongoing pandemic.
Joshi et al^81^	2022	Respiratory ECMO Survival Prediction (RESP) Score for COVID-19 Patients Treated with ECMO	The objective of this study is to assess the effectiveness of the RESP score in predicting the survival rate of COVID-19 patients receiving VV-ECMO treatment during their hospital stay.

ECMO, extracorporeal membrane oxygenation; ELSO, Extracorporeal Life Support Organization; RESP, respiratory ECMO survival prediction; VV-ECMO, veno-venous extracorporeal membrane oxygenation.
